# Editorial: Epigenetics in Plant Development

**DOI:** 10.3389/fpls.2022.864945

**Published:** 2022-02-28

**Authors:** Nobutoshi Yamaguchi

**Affiliations:** Division of Biological Science, Graduate School of Science and Technology, Nara Institute of Science and Technology, Ikoma, Japan

**Keywords:** chromatin structure, DNA methylation, development, epigenetics, environmental response, histone modification

Plant growth and development are determined by the spatiotemporal regulation of gene expression and epigenetic regulators help fine-tune the timing and patterns of gene expression. For example, as a part of this Research Topic on Epigenetics in Plant Development, Yamaguchi describes recent findings about one of the best-characterized plant transcription factors, LEAFY (LFY), in *Arabidopsis*. Although many researchers have examined LFY function over the past 30 years, two independent research groups recently revealed that LFY functions as a pioneer transcription factor, one of the master regulators located at the top of the gene regulatory hierarchy. Pioneer transcription factors reprogram the closed chromatin of their target genes and thus play critical roles in specifying when and where downstream targets are expressed to ensure proper cell fate and differentiation. LFY directly binds condensed chromatin, displaces the linker histone H1 in the nucleosome, interacts with chromatin remodeling factors, and opens up chromatin to enable the binding of other factors to specify floral fate (Weigel et al., 1992; Jin et al., 2021; Lai et al., 2021). The emerging research on LFY is just one example of recent major breakthroughs in this field, but much more remains to be learned about the epigenetic mechanisms underlying plant development.

Epigenetic regulation involves multiple mechanisms, including histone modifications. Fang et al. highlight the role of histone H3 lysine methylation in regulating gene expression, with extra emphasis on reproductive development in *Arabidopsis*. Members of the SET Domain Group (SDG) serve as “writers” by depositing methylation marks (Pontvianne et al., 2010). Histone marks are recognized by “readers,” such as proteins with PHD domains, WD40 repeats, and Chromo domains (Jiang et al., 2009). By contrast, LYSINE-SPECIFIC DEMETHYLASE 1 (LSD1) and Jumonji-C domain-containing proteins (JMJs) remove methylation marks, thus serving as “erasers.” Yamaguchi focuses on a group of *Arabidopsis* JMJ proteins that remove trimethylation of histone H3 lysine 27 (H3K27me3). The H3K27me3 demethylases identified to date include EARLY FLOWERING 6 (ELF6)/JMJ11, RELATIVE OF ELF6 (REF6)/JMJ12, JMJ13, JMJ30, and JMJ32. These proteins often function in a redundant manner to regulate plant development and environmental responses. Keyzor et al., studied the relationship between ELF6 and JMJ13 and revealed their antagonistic functions during *Arabidopsis* flower development. Compared to the wild type, *elf6* displays increased self-fertility, whereas *jmj13* mutants show decreased self-fertility. Based on transcription data, ELF6 promotes carpel elongation by activating expansin genes. JMJ13 represses carpel growth by activating jasmonic acid signal transduction and promotes stamen growth by activating *SAUR26* expression.

Each epigenetic factor can play multiple roles in controlling gene expression in a tissue-specific manner. Ornelas-Ayala et al., introduce multiple interacting partners of ULTRAPETALA1 (ULT1) in *Arabidopsis*. ULT1 controls histone H3 lysine 4 (H3K4me3) levels and counteracts the activity of H3K27me3 “writers.” ULT1 physically interacts with the H3K4me3 writer ATX1 to induce H3K4me3 deposition, and it interacts with tissue-specific transcription factors. ULT and the GARP family transcription factor KANADI1 (KAN1) form a complex that controls gynoecium axis development. ULT and the MYB domain-containing transcription factor ULTRAPETALA INTERACTING FACTOR 1 (UIF1) control floral meristem determinacy by repressing the expression of the stem cell fate gene *WUSCHEL*. Chromatin structure is altered by chromatin remodelers, such as ATP-dependent chromatin remodeling SWITCH/SUCROSE NON-FERMENTING (SWI/SNF) complexes. The SWI/SNF complex component SWI3B was initially identified as a flowering time regulator (Sarnowski et al., 2002). Lin et al., identified a new interacting partner for SWI3B. SWI3B genetically and physically interacts with LEAF AND FLOWER RELATED (LFR) to determine adaxial-abaxial cell fate in leaves.

In addition to our knowledge of interacting partners, the factors that function upstream and downstream of each epigenetic factor are not fully understood. Jiang and Zheng summarize the current understanding of the relationship between SPOROCYTELESS/NOZZLE (the core transcription factor required for megaspore mother cell development) and epigenetic regulation at multiple layers. Hirai et al., explored factors downstream of histone deacetylase activity during xylem vessel cell differentiation and identified *OVATE FAMILY PROTEIN1* (*OFP1*), *OFP4*, and *MYB75* as downstream targets. These genes encode transcription factors that form a complex with BEL1-LIKE HOMEODOMAIN6 to control gene expression for cell differentiation.

Although the majority of reviews and research articles in this Research Topic describe work in *Arabidopsis* due to the relative ease in performing epigenetic analysis in this plant, a few researchers have performed epigenetic studies in other plant species. Zhang et al., obtained genome-wide H3K27me3 and H3K4me3 profiles in allotetraploid cotton (*Gossypium hirsutum*). In general, H3K4me3 and H3K27me3 are located around the transcription start sites of active genes and the gene bodies of silenced genes, respectively. Consistent with this notion, the presence of H3K4me3 and H3K27me3 leads to the activation and repression of gene expression, respectively, in allotetraploid cotton. Examining the roles of histone-modifying enzymes in other plant species remains an exciting area for future research.

Many studies related to this Research Topic have revealed the importance of epigenetic regulation in cell fate switching or developmental transitions. These processes occur in a limited number of cells during a limited time window. However, techniques such as chromatin immunoprecipitation followed by sequencing (ChIP-seq) require large numbers of cells and take several days to perform. To address these problems, Ouyang et al., developed an alternative method for ChIP-seq called nucleus CUT&Tag (nCUT&Tag). nCUT&Tag can be completed within a day using only 0.01 g of plant tissue as the starting material. Cao et al., explored DNA methylation dynamics using a tissue culture system to prepare plant materials at different stages of development. The combination of such sophisticated systems and highly sensitive techniques will allow researchers to further explore the epigenetic regulation of gene expression during plant development in the future.

## Author Contributions

The author confirms being the sole contributor of this work and has approved it for publication.

## Funding

This work was supported by a grant from the Japan Science and Technology Agency PREST(JPMJPR15QA), a JSPS KAKENHI Grant-in-Aid for Scientific Research on Innovative Areas (No. 18H04782), a JSPS KAKENHI Grant-in-Aid for Scientific Research B (No. 18H02465), a Grant-in-Aid for challenging Exploratory Research (No. 19K22431), and a grant from the SECOM Science and Technology Foundation to NY.

## Conflict of Interest

The author declares that the research was conducted in the absence of any commercial or financial relationships that could be construed as a potential conflict of interest.

## Publisher's Note

All claims expressed in this article are solely those of the authors and do not necessarily represent those of their affiliated organizations, or those of the publisher, the editors and the reviewers. Any product that may be evaluated in this article, or claim that may be made by its manufacturer, is not guaranteed or endorsed by the publisher.
